# Specific targeting of whole lymphoma cells to dendritic cells *ex vivo *provides a potent antitumor vaccine

**DOI:** 10.1186/1479-5876-5-16

**Published:** 2007-03-14

**Authors:** Christian Adam, Josef Mysliwietz, Ralph Mocikat

**Affiliations:** 1 GSF-Institut für Molekulare Immunologie, Marchioninistr. 25, 81377 München, Germany

## Abstract

**Background:**

Dendritic cells (DC) pulsed with tumor-derived antigenic material have widely been used in antitumor vaccination protocols. However, the optimal strategy of DC loading has not yet been established. Our aim was to define requirements of optimal DC vaccines in terms of *in vivo *protection in a murine B-cell lymphoma model.

**Methods:**

We compare various loading reagents including whole parental and modified tumor cells and a single tumor-specific antigen, namely the lymphoma idiotype (Id). Bone marrow-derived DC were pulsed *in vitro *and used for therapy of established A20 lymphomas.

**Results:**

We show that a vaccine with superior antitumor efficacy can be generated when DC are loaded with whole modified tumor cells which provide both (i) antigenic polyvalency and (ii) receptor-mediated antigen internalization. Uptake of cellular material was greatly enhanced when the tumor cells used for DC pulsing were engineered to express an anti-Fc receptor immunoglobulin specificity. Upon transfer of these DC, established tumor burdens were eradicated in 50% of mice. By contrast, pulsing DC with unmodified lymphoma cells or with the lymphoma Id, even when it was endowed with the anti-Fc receptor binding arm, was far less effective. A specific humoral anti-Id response could be detected, particularly following delivery of Id protein-pulsed DC, but it was not predictive of tumor protection. Instead a T-cell response was pivotal for successful tumor protection. Interaction of the transferred DC with CD8^+ ^T lymphocytes seemed to play a role for induction of the immune response but was dispensable when DC had received an additional maturation stimulus.

**Conclusion:**

Our analyses show that the advantages of specific antigen redirection and antigenic polyvalency can be combined to generate DC-based vaccines with superior antitumor efficacy. This mouse model may provide information for the standardization of DC-based vaccination protocols.

## Background

Dendritic cells (DC) are professional antigen-presenting cells (APC) that are effective at presenting immunogenic peptides in the context of major histocompatibility complex (MHC) molecules and providing the costimulatory signals necessary for efficient T-cell stimulation [1-3]. DC-based vaccination against tumor-associated antigens (TAA) has received much interest in experimental cancer therapy. To allow TAA to be efficiently presented to T cells, DC were pulsed with proteins or synthetic peptides [4,5] or transduced with viral vectors [6,7]. In murine tumor models, DC loaded with single TAA induced effective antitumor responses [5,8]. However, vaccination with a single antigen (Ag) may be less efficient than immunization against a panel of Ag because in the latter setting, selection of Ag loss mutants is less likely to occur and even still unidentified TAA can be included [9-13]. DC were therefore used in tumor vaccination protocols that were pulsed with whole tumor cell lysates [14]. On the other hand, it was shown that loading of more than one epitope on APC inhibits T-cell priming [15] due to competition of peptides for MHC molecules and of T cells for access to APC. The consequences of this finding for antitumor immunization *in vivo*, however, have not yet been addressed and the optimal DC loading strategy still remains to be defined.

A critical step in antitumor immunization is the engulfment of TAA by APC. It was shown that this is most efficiently mediated by adsorptive endocytosis. Thus, antibody- (Ab-) dependent redirection of Ag to endocytosing Fc receptors FcγRI and FcγRII expressed by APC gave rise to potent immune responses [16-25]. Induction of Ag-specific humoral responses *in vivo *was even possible against weak immunogens such as the immunoglobulin (Ig) idiotype (Id) of B-cell lymphomas [9,12,25] which is an absolutely tumor-specific Ag. However, this immunity was not capable of conferring complete tumor protection in some mouse models *in vivo *[9,12]. As immunization against a panel of tumor-derived Ag turned out to be necessary for successful tumor rejection in different tumor models [9-13,26], we investigated exogenous loading of DC that relies on Ag targeting to FcγR but nevertheless can provide antigenic polyvalency. Both antigenic polyvalency and receptor-mediated Ag internalization was achieved by pulsing DC with whole tumor cells that were modified to express an FcγR specificity.

Tumor rejection *in vivo *is the most rigorous readout system for antitumor immunity. A systematic comparison revealed that in terms of tumor therapy, whole anti-FcγR-expressing tumor cells were most suitable for DC loading not only in comparison to unmodified lymphoma cells but also to soluble Id proteins even when these were engineered to be redirected to FcγR. This system showed that a possible competition of multiple epitopes as described elsewhere [15] plays no role for successful tumor rejection *in vivo*.

## Methods

### Cell culture

All cells were cultured in RPMI 1640 medium supplemented with 5% fetal calf serum, 2 mM glutamine, nonessential amino acids, 50 μM 2-mercaptoethanol and antibiotics at 37°C in a humidified 5% CO_2 _atmosphere. The trioma cell line BiV was generated by fusing A20 cells [27] to the rat anti-mouse FcγR hybridoma 2.4G2 as described earlier [9]. 5C12 is an A20 variant that was genetically engineered to express the Id as an Ig/GM-CSF fusion protein [10]. DC were prepared by culturing bone marrow precursors from BALB/c wildtype or β_2_m^-/- ^mice in standard medium in the presence of 100 ng/ml recombinant murine granulocyte-macrophage colony-stimulating factor (GM-CSF) [26]. Medium was replaced every two days. On day 8, DC were pulsed for 12 hours with A20 or BiV cells (irradiated at 100 Gy and 20 Gy, respectively; cell ratio 1:1) or with 100 to 300 μg/ml A20 Id, BiV heterodimeric Ig protein or with 5C12 fusion protein. The latter was used for differentiation of DC from bone marrow precursors as well as for the loading step. The proteins were purified from culture supernatants using protein A chromatography as described elsewhere [9,10,28]. If not otherwise indicated, DC were then exposed to 1 μg/ml lipopolysaccharide (LPS) for maturation. DC were extensively characterized by fluorescence-activated cell sorting (FACS) using monoclonal Ab (mAb) against MHC class I, MHC class II, CD80, CD86 and CD40. Prior to injection into mice, DC were separated from the loading proteins by washing and from the cellular Ag sources by negative immunomagnetic separation (Miltenyi Biotec, Bergisch-Gladbach, Germany) using the anti-Id mAb 6C10 [10]. Control experiments showed that BiV cells alone which were depleted by this separation step had no therapeutic effect.

### Cell uptake assay and confocal microscopy

BiV and A20 cells were irradiated or killed by one freeze-thaw cycle and labelled with 10 μM per 10^7 ^cells of carboxyfluorescein diacetate succinimidyl ester (CFDA; Invitrogen, Karlsruhe, Germany) as recommended by the manufacturer. Immature DC (iDC) were harvested on day 8 and coincubated with the CFDA-labelled A20 or BiV cells for 1 or 4 hr at 37°C or 4°C. Then DC were labelled with PE-conjugated anti-CD11c or anti-CD80 mAb (Pharmingen, Heidelberg, Germany) and Cy5-conjugated anti-MHC class II (HB3, ATCC, Manassas, USA). Uptake of CFDA-labelled A20 or BiV cells was monitored using multicolour flow cytometry (CyAn-ADP, Dakocytomation, Hamburg, Germany) by gating of CD11c/MHCII double-positive DC. Dead cells were excluded using propidium iodide vital staining.

For confocal microscopy, DC that had ingested CFDA-labelled cells as described above were counterstained with TRITC-conjugated anti-CD11c. The DC were then attached to poly-L-lysin-coated slides as described previously [29], fixed for 20 min. using 2% paraformaldehyde and processed on a Leica TCS SP2 confocal microscope. The cell uptake and microscopic studies were performed three to five times.

### Animal experiments

BALB/c wildtype and β_2_m^-/- ^mice were purchased from Bommice (Ry, Denmark) and The Jackson Laboratories (Bar Harbor, USA), respectively. All animal experiments were approved by the *Regierung von Oberbayern*. Groups of 6 female mice received a lethal tumor challenge i.v. (7 to 8 × 10^5 ^A20 cells) at day 1. Therapy was done by s.c. injection of 5 × 10^5 ^DC at day 5 and again at day 11. To assess longterm memory, surviving animals received an A20 rechallenge about 100 days later. Mice were killed when first signs of tumor growth appeared. Depletion of T-cell subsets was done by injecting mAb RmCD4-2 or RmCD8-2 i.p. starting 4 days before therapy [9]. To test for humoral immune responses mice were bled before beginning of the experiment and three weeks after therapy. All animal experiments were performed at least twice. In the figures, typical results are shown. Statistical analyses were done using the logrank test. Survival curves are shown until day 200 although the animals were followed at least for 1 1/2 years. Generally, mice surviving day 100 remained tumor-free life-long.

### T-cell proliferation assay and test for humoral anti-Id immunity

After pulsing and maturation, DC were irradiated at 20 Gy and cocultivated with syngeneic T cells at different T cell/DC ratios in 96-well round-bottom plates. 5 days later, T cells were pulsed with 1 μCi ^3^H-thymidine. Cells were harvested after 20 hours and incorporated radioactivity was determined in a liquid scintillation counter. Proliferation indices were calculated by normalizing the radioactivity incorporated in T cells to the background levels without T cells. Mean indices and standard deviations were calculated from three experiments.

Anti-Id responses in mouse sera were determined by enzyme-linked immunosorbent assay (ELISA) exactly as described previously [9,10]. In brief, Ab in mouse sera were captured by the A20 Ig purified from culture supernatants and detected by polyclonal anti-mouse Fc Ab which was preabsorbed against mouse IgG2a. Results were expressed as those reciprocal Ab titres that gave two-fold extinction above background.

## Results and Discussion

### Loading of DC *in vitro*

**Figure 1:**
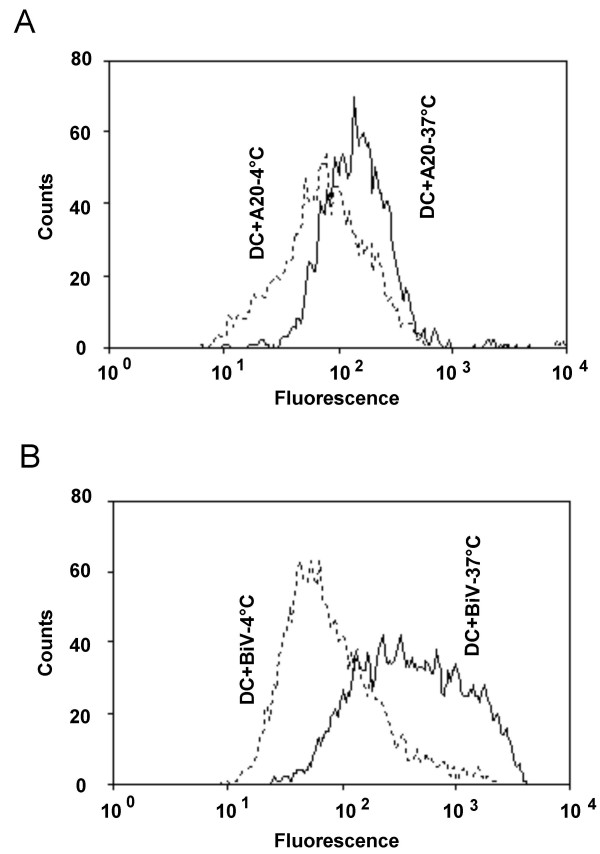
**Incorporation of cellular material derived from unmodified lymphoma cells or from trioma cells by DC**. A20 (a) or BiV cells (b) were labelled with CFDA and coincubated for 1 hour with iDC at 4°C or 37°C. CFDA fluorescence of viable, CD11c and MHC class II double-positive DC was measured by flow cytometry. Typical result from 5 experiments using BiV and 3 experiments using A20 cells. The average uptake of A20 cells was 1.5-fold increased at 37°C in comparison to 4°C (standard deviation = 0.2), the internalization of BiV cells was increased 8.5-fold in an average (s = 1.2).

**Figure 2:**
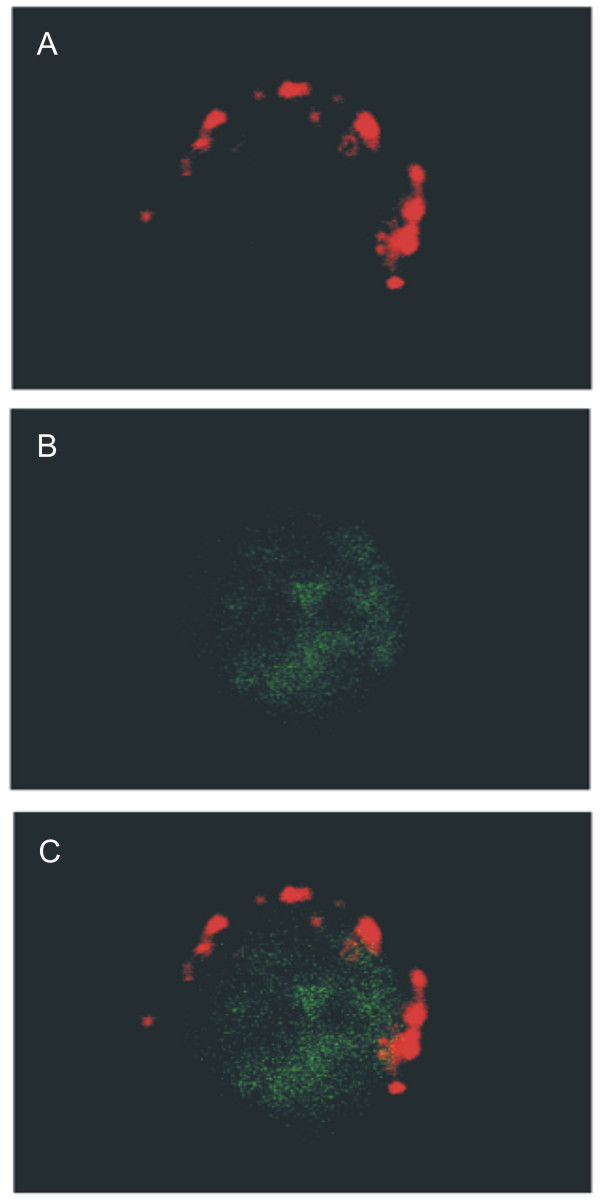
**Confocal microscopy of DC after internalization of CFDA-labelled A20 cells**. Uptake of cells was performed for 1 hr at 37°C. (a) DC visualized with TRITC-conjugated anti-CD11c Ab (red). (b) CFDA fluorescence. (c) Overlay of TRITC and CFDA fluorescence. Typical result from 3 independent experiments.

The murine A20 lymphoma was converted to the trioma cell line BiV by fusion with a xenogeneic (rat) hybridoma producing an anti-mouse FcγR Ab. The trioma cells contained most if not all genetic material derived from A20 [12] and expressed the anti-APC specificity as secreted and as membrane-bound Ab [9,12]. It is established that targeting of soluble Ag to FcγR expressed on APC induces potent immune responses. To show that even a variety of TAA can be efficiently redirected to APC *in vitro *when whole cells are endowed with the anti-FcγR specificity, iDC were cultured with irradiated CFDA-labelled A20 or BiV cells. After one hour of coincubation at 4°C or at 37°C, the fluorescence of the DC was measured by flow cytometry. While the average uptake of A20 cells was only 1.5-fold enhanced at 37°C in comparison to 4°C (Fig. 1a), the internalization of BiV cells was increased 8.5-fold (Fig. 1b), in some experiments up to 12-fold. Identical results were obtained after 1 hour and after 4 hours of coincubation. To show that the data reflect ingestion of the cells by the DC and not only aggregation of cells or membrane fragments to the surface of DC, confocal microscopy was performed. As exemplarily shown in Fig. 2c, labelled fragments were indeed detected intracellularly as can be seen by overlay of CD11c (Fig. 2a) and CFDA (Fig. 2b) staining.

**Figure 3:**
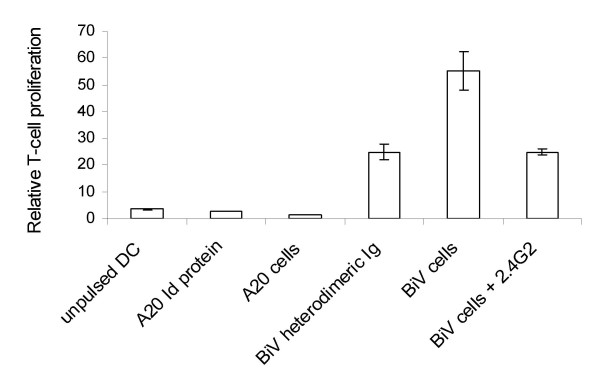
**T-cell proliferation after priming with DC that were pulsed with the indicated reagents**. DC were unpulsed or loaded with the soluble A20 Ig idiotype, with A20 cells, with a soluble heterodimeric A20 Ig idiotype containing the anti-FcR binding arm or with BiV cells. For blocking FcR-mediated Ag uptake during coincubation with BiV cells, the anti-FcR mAb 2.4G2 was added. Proliferation of T cells was determined by ^3^H-thymidine uptake. Proliferation indices were calculated by normalizing the radioactivity incorporated in T cells to the background levels without T cells. The bars represent results at a T cell/DC ratio of 9:1. Mean values and standard deviations are compiled from 3 experiments.

We then tested the consequences of DC loading for stimulating syngeneic T lymphocytes in proliferation assays (Fig. 3). The stimulating potential of DC that were pulsed with irradiated A20 cells or with the purified A20 Ig did not differ from unpulsed DC. To assess the effect of FcγR-targeting of the A20 Ig we used a construct which combines the murine lymphoma Id with the rat anti-APC binding arm in the form of a heterodimeric Ig. Due to preferential pairing between Ig heavy and light chains of corresponding specificities observed when the parental antibodies are of different species origin [28], the BiV cells expressed this "bispecific Ab" at high yield and also secreted it into the culture supernatant [9]. In contrast to the unmodified purified Id protein, the soluble A20/anti-APC Ig heterodimer when loaded to DC gave rise to a clear T-cell response which might be directed against the A20 Id but also to xenogeneic determinants derived from the rat Ig. Apart from enhanced Ag engulfment mediated by FcR-targeting, a higher stimulatory capacity of FcR-stimulated DC may promote the effect, although alterations of maturation markers were not seen after incubation of DC with anti-FcR. When whole irradiated BiV cells were used for DC pulsing, the T-cell response was most pronounced. The additional increase as compared to the heterodimeric Ig protein may be explained by the antigenic polyvalency provided by whole cells. T cells will be directed against multiple epitopes derived from the tumor cells as well as against a variety of xenogeneic peptides derived from the fusion partner of the trioma. Of course, it is not clear from the stimulation experiments what the extent of an antitumor and the xenogeneic response may be, but *in vivo *experiments clearly showed that there was indeed a tumor-reactive component (*vide infra*). As predicted, blocking FcγR by the soluble anti-FcγR mAb 2.4G2 during cocultivation reduced the T-cell-stimulating activity of the DC (Fig. 3). The T-cell proliferation remaining after blocking with 2.4G2 could be related to receptor-independent Ag uptake and xeno-reactivity, but it is also possible that mAb blocking was not complete. In summary, the superior T-cell-activating effect exerted by trioma cell-pulsed DC may be due (i) to the enhanced FcγR-mediated Ag internalization, (ii) to the antigenic polyvalency, (iii) to the xenogeneic moiety included in the triomas and (iv) possibly to an increased stimulatory capacity of FcR-trigerred DC.

### Therapeutic effect of trioma-loaded DC

**Figure 4:**
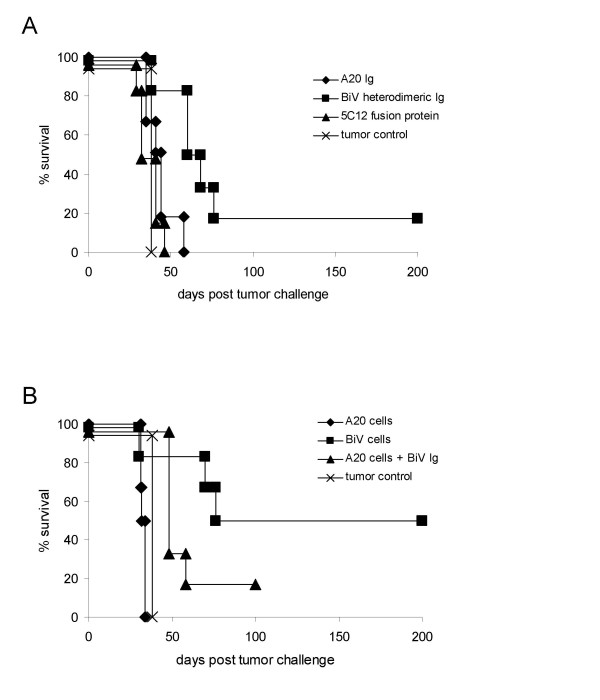
**Eradication of established A20 lymphomas by DC that were pulsed with the indicated reagents**. (a) Effect of loading DC with different formulations of the purified soluble A20 Id. (b) Effect of loading DC with whole unmodified lymphoma cells (A20) or with trioma cells (BiV) or with a mixture of A20 cells and purified BiV heterodimeric Ig. The difference between the trioma cell-treated group and the control group without therapy was significant with p < 0.05 (logrank test).

To examine the *in vivo *consequences of T-cell activation, DC that were pulsed with various tumor-derived reagents were used for the therapy of established A20 burdens in mice. Whereas DC loaded with unmodified Id had no therapeutic effect, those DC that were pulsed with the heterodimeric Ig derived from BiV were able to confer a modest survival benefit (Fig. 4a). Because GM-CSF receptors are also thought to mediate Ag internalization, we also delivered the A20 Id to DC in form of an Ig/GM-CSF fusion protein. The construct 5C12 which contains GM-CSF attached to the Fc region of the A20 Ig [10] induced differentiation of DC (not shown), but those DC whose GM-CSF receptors were targeted were not able to rescue mice from tumor growth (Fig. 4a).

We then examined the therapeutic potential of DC that were pulsed *in vitro *with the cellular Ag sources. Loading with irradiated A20 cells was ineffective, while 50% of tumor-bearing mice were cured upon transfer of BiV cell-pulsed DC (Fig. 4b). These animals also developed a longterm memory because they were able to reject a wildtype tumor rechallenge given about 100 days later (not shown). Pulsing DC with a mixture of A20 cells and the soluble heterodimeric Id protein had a similar marginal effect as the Id protein alone (Fig. 4b). Thus, it is pivotal that the anti-FcγR specificity be expressed by the cellular Ag source. The advantage of the cellular vaccine can only be exploited in combination with its anti-FcγR expression. The rejection of the wildtype A20 lymphoma *in vivo *exactly reflected the hierarchy of T-cell activation observed in the proliferation assays. Whereas the *in vitro *data did not allow to distinguish between tumor-specific and xeno-reactive T-cell activation mediated by BiV cell-pulsed DC, the therapy studies demonstrated that, *in vivo*, T cells were activated which are indeed tumor- and not only xeno-reactive. In summary, we assume that a prerequisite for successful antitumor therapy is the combination of FcγR redirection and antigenic polyvalency. T cells stimulated by xenogeneic peptides should additionally enhance the antitumor efficacy by virtue of a bystander effect [9,12].

### Mechanisms of DC-induced tumor immunity

**Figure 5:**
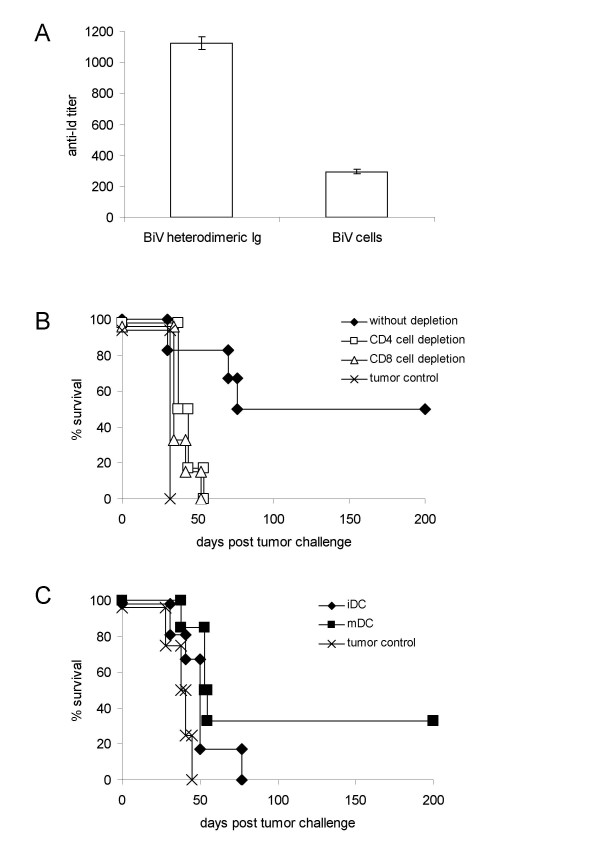
**Mechanisms of DC-mediated tumor protection**. (a) Humoral anti-Id responses in mice vaccinated with DC that were pulsed with the purified A20 Id/anti-FcγR heterodimeric BiV protein or with whole BiV cells. Results were expressed as those reciprocal Ab titres that gave two-fold extinction above background. Means were calculated from 6 (BiV protein) and from 12 mice (BiV cells), respectively. Standard deviations are also indicated. (b) Effect of CD4^+ ^and CD8^+ ^T-cell depletion on survival of mice treated with DC that were pulsed with BiV cells. The survival of CD4^+ ^or CD8^+ ^T-cell-depleted mice was not significantly different from the tumor control group that did not receive any therapy (logrank test). (c) Survival of tumor-bearing mice that were injected with trioma cell-loaded DC from β_2_m-deficient animals which were or were not matured by LPS following the loading step. The therapeutic effect of BiV cell-pulsed mDC from β_2_m-deficient animals was not significantly different from BiV cell-pulsed mDC from normal mice (logrank test). Tumor control groups without therapy are also shown in both survival diagrams. Typical results are depicted from two experiments in each setting.

Since the Id is a unique tumor Ag expressed on B-cell lymphoma we tested for the presence of humoral anti-Id responses in mice having received therapy with DC that were loaded with BiV protein or BiV cells. There were considerably higher anti-Id titres when DC were loaded with the specific protein (Fig. 5a) although tumor protection was less efficient. Furthermore, Ab responses in mice that received BiV cell-pulsed DC did not correlate with the individual survival times. Thus, the advantage of pulsing DC with whole trioma cells which is clearly demonstrated by the survival benefit is not reflected by the Id-specific Ab titres. *In vivo *protection may be dependent on other specificities than the Id or exclusively on a cellular immune response. Nonetheless, a role of cytotoxic anti-Id Ab for mediating tumor eradication cannot be completely excluded.

To elucidate the role of the cellular antitumor response mice were depleted of T cells before the onset of therapy. Ablation of CD4^+ ^cells or CD8^+ ^cells totally abrogated the therapeutic effect of trioma cell-pulsed DC (Fig. 5b). This indicates that both T-cell subsets are crucial for DC-induced tumor immunity. The requirement for CD4^+ ^T lymphocytes may be in accordance with the concept that exogenous proteins are taken up and processed by DC and presented to CD4^+ ^cells in association with MHC class II molecules. Activated CD4^+ ^cells then provide help for efficient generation of CTL. In contrast, intracellular Ag are thought to be presented by MHC class I molecules to CD8^+ ^CTL, but it has become clear that exogenous proteins can also be directed to the endogenous presentation pathway thus leading to CTL induction through cross-presentation.

To answer the question whether DC whose FcγR are targeted with trioma cells only present immunogenic peptides to CD4^+ ^cells or cross-presentation to CD8^+ ^cells also plays a role, therapy experiments were repeated using trioma cell-pulsed DC from β_2_m-deficient mice. When these DC were transferred in an immature state, therapy failed (Fig. 5c) whereas iDC from normal mice were effective. As β_2_m-deficient DC cannot directly interact with CD8^+ ^T lymphocytes, we conclude that cross-presentation to CD8^+ ^cells is necessary for inducing optimal tumor protection in this setting. In contrast, when β_2_m-deficient DC were subjected to a maturation step following pulsing with trioma cells, the therapeutic effect was not significantly different from the effect which was observed using mDC from wildtype mice. It is conceivable that mDC circumvent the need of direct contact between transferred DC and CD8^+ ^T cells because they activate helper cells more efficiently than their immature counterparts.

## Conclusion

Trioma cells are B-cell lymphomas modified to express an FcγR binding specificity [9]. By targeting whole trioma cells that harbour potentially all lymphoma-derived Ag against FcγR-bearing professional APC, this approach has the potential to overcome the insufficient Ag presentation by malignant B cells and to induce a polyvalent antitumor response, as was shown by delivering trioma cells as a cellular vaccine *in vivo *[9,12,26]. In the present study, we set out to optimize exogenous pulsing of DC for tumor immunotherapy in a murine lymphoma model and established an *ex vivo *modification of the trioma approach. DC for vaccination against B-cell lymphoma were pulsed with trioma cells thereby combining antigenic polyvalency and targeting of tumor-derived Ag to FcγR expressed on DC. Other endocytic receptors like DEC-205 may also be suitable target structures for directing Ag to DC [30]. Xenogeneic peptides presented by DC which have been pulsed with trioma cells are likely to further enhance T-cell activation.

As a model for a single tumor Ag, we included in our analyses the lymphoma Id which has been considered as an attractive tumor rejection target because it is an absolutely unique tumor Ag. *In vitro *studies and clinical investigations revealed the induction of specific CTL responses by Id-pulsed DC [31-33]. In the A20 model investigated in our study, however, DC loaded with Id protein alone, even in the form of an Id/anti-FcγR heterodimer, exerted only a marginal therapeutic effect *in vivo*. This may be explained by the A20 tumor being a highly aggressive B-cell lymphoma. The successful therapy of pre-established, disseminated tumor burdens using trioma cell-based DC therefore highlights the potency of this approach. Although Id-specific humoral responses could be detected after redirecting *ex vivo *to FcγR the Id protein or whole trioma cells, these Id-specific Ab titres never correlated with tumor protection *in vivo*. Rather, T cells were instrumental for tumor eradication.

Our results show that a possible competition between different Ag [15] does not hamper the *in vivo *effect. Of course, we cannot preclude a selection of Ag and a bias towards peptides of high affinity, but the peptides that are presented in the context of MHC molecules and give rise to T-cell activation seem to be sufficient to induce tumor eradication.

As malignant lymphoma in humans still has a poor prognosis, new therapeutic modalities are needed. The mouse model explored in this paper may give hints to the development of DC-based immunotherapies of malignant Non-Hodgkin lymphoma.

## Competing interests

The author(s) declare that they have no competing interests.

## Authors' contributions

CA carried out all experiments except cell uptake assays and confocal microscopy. JM performed cell uptake assays and confocal microscopy and participated in drafting the manuscript and preparing the figures. RM conceived, designed and coordinated the study and drafted the manuscript. All authors read and approved the final manuscript.
